# Supplementary figures and images for: Fast and robust estimate of bacterial genus novelty using the percentage of conserved proteins with unique matches (POCPu)
Source: PeerJ. 2025 Nov 14;13:e20259. doi: 10.7717/peerj.20259 (PMC12622232; doi:10.7717/peerj.20259)

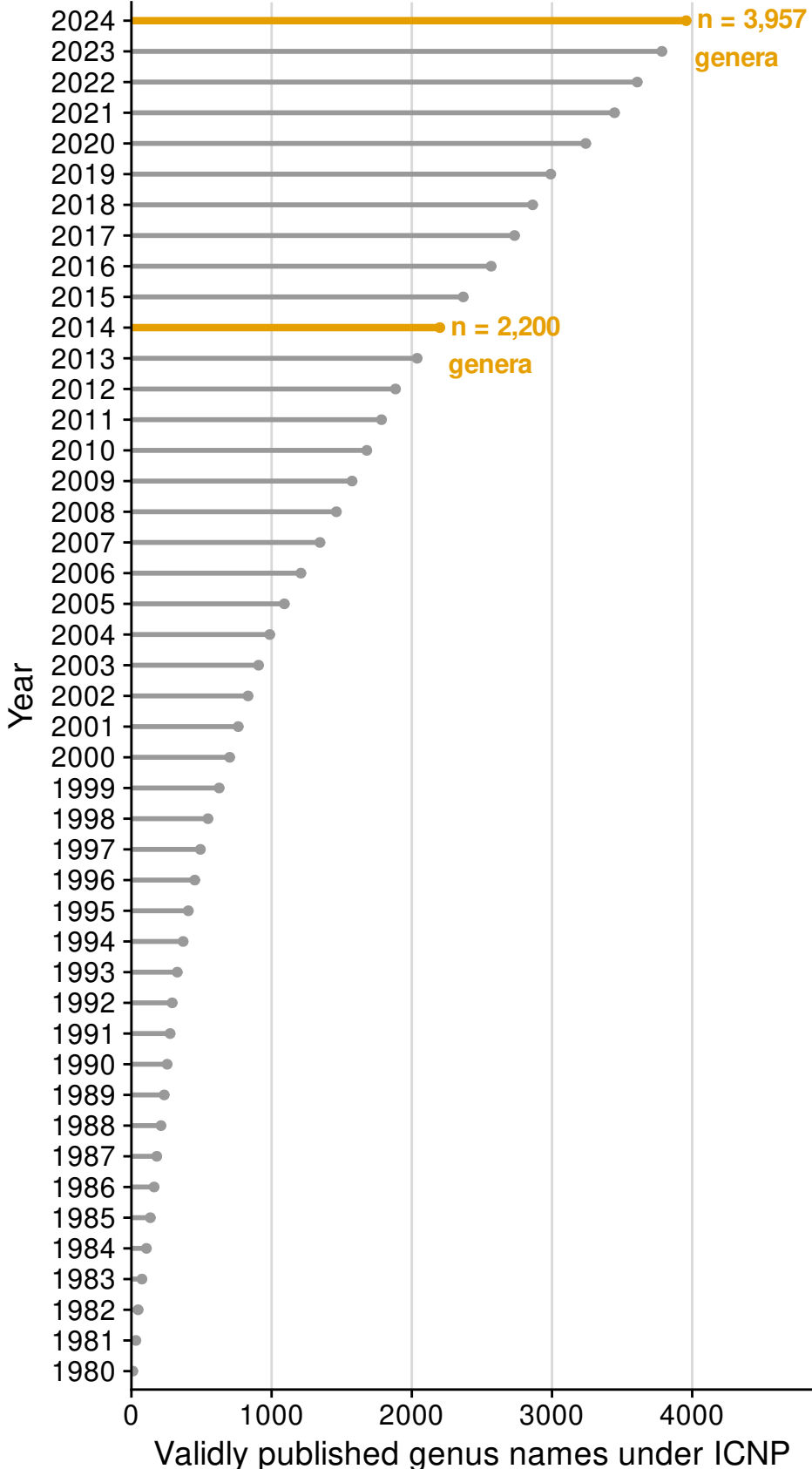

Supplement: Supplemental Information 4 — The year 2014 is highlighted as it corresponds to the year of publication of the paper by Qin et al. (2014) describing the Percentage of Conserved Proteins (POCP) to delineate genus. The number of valid genera is highlighted ten years later. The data was accessed on 2024-12-11 at the List of Prokaryotic names with Standing in Nomenclature (Parte et al., 2020). [file peerj-13-20259-s004.pdf]

POCP based on BLAST\_BLASTPDB (in %)

$R^2 = 1$   
 $p < 0.001$

POCP based on BLAST\_BLASTP (in %)

Data points per hexagon

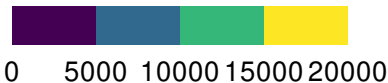

Supplement: Supplemental Information 5 — Each point (n = 70,602) represents a POCP value between two genomes (see (1)). The colors represent the number of data points binned together in hexagons to avoid over-plotting. Coefficient of determination (R 2) and associated p-value are shown on top of each linear regressions. [file peerj-13-20259-s005.pdf]

POCPu based on BLAST\_BLASTPDB (in %)

$R^2 = 1$   
 $p < 0.001$

POCPu based on BLAST\_BLASTP (in %)

Data points per hexagon

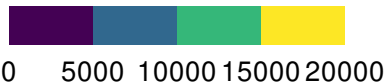

Supplement: Supplemental Information 6 — Each point (n = 70,602) represents a POCPu value between two genomes (see (2)). The colors represent the number of data points binned together in hexagons to avoid over-plotting. Coefficient of determination (R 2) and associated p-value are shown on top of each linear regressions. [file peerj-13-20259-s006.pdf]

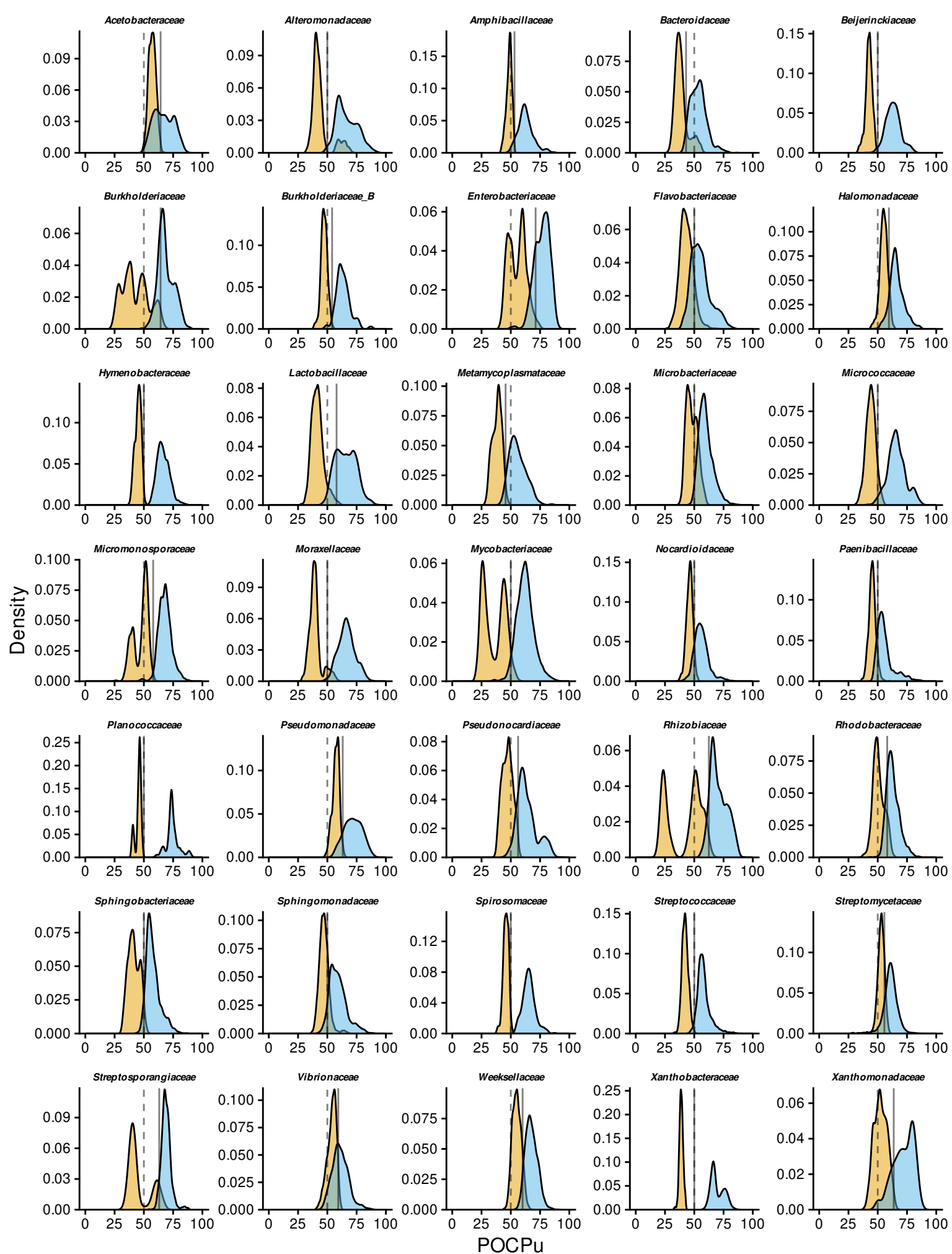

Supplement: Supplemental Information 7 — The true category is based on the GTDB taxonomy. The family-specific POCPu thresholds for genus delineation proposed in this study were taken from Table 2 and are indicated with plain vertical line, whilst the default POCPu threshold of 50% is indicated by dashed lines. [file peerj-13-20259-s007.pdf]

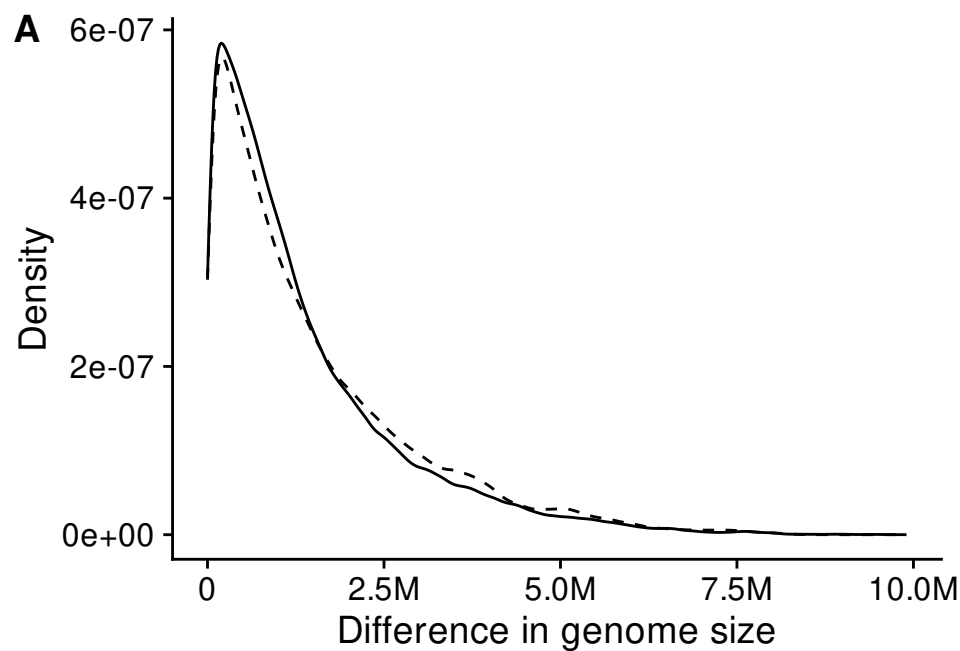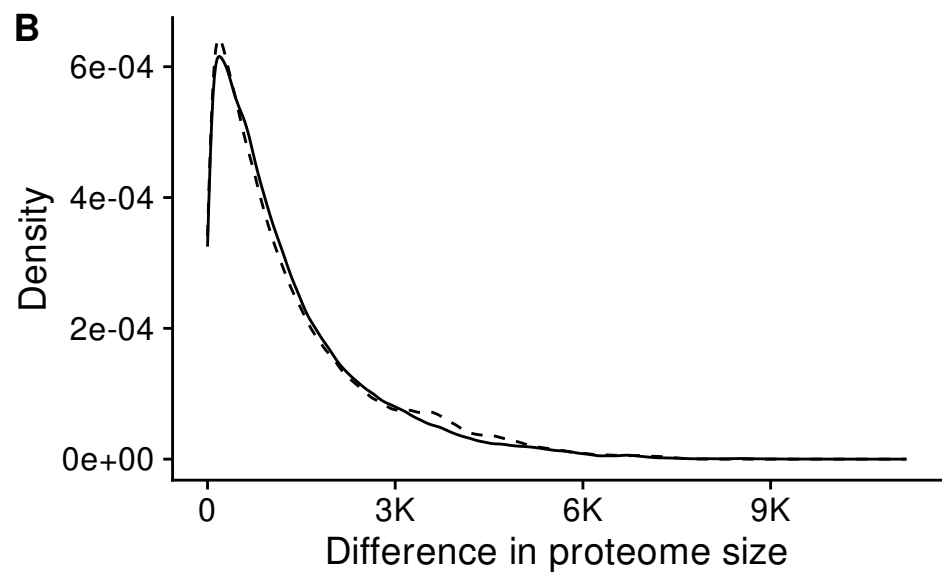

POCPu Threshold [---] Default [—] Optimized

Supplement: Supplemental Information 8 — Distributions of differences in genome size (A) and proteome size (B) for families using default threshold or optimized thresholds. In case of an association between genome (or proteome) size and POCPu, we expected families for which optimized thresholds are proposed to have a shift towards larger differences explaining poor delineation performance in Fig. 5B. This was not the case, indicating that genome size and proteome size did not influence genus delineation. POCPu thresholds type were taken from Table 2. [file peerj-13-20259-s008.pdf]
