# Supplementary material for: Fast and robust estimate of bacterial genus novelty using the percentage of conserved proteins with unique matches (POCPu)
Source: PeerJ. 2025 Nov 14;13:e20259. doi: 10.7717/peerj.20259 (PMC12622232; doi:10.7717/peerj.20259)
Supplement: Supplementary file 5 [file peerj-13-20259-s002.odt]

| Method | Database creation | Parameters |
| --- | --- | --- |
| BLAST |
| BLASTP | No | --evalue 0.00001 --qcov_hsp_perc 50.0 |
| BLASTPDB | Yes | --evalue 0.00001 --qcov_hsp_perc 50.0 |
| DIAMOND |
| DIAMOND_FAST | Yes | --evalue 0.00001 --query-cover 50.0 --fast |
| DIAMOND_SENSITIVE | Yes | --evalue 0.00001 --query-cover 50.0 --sensitive |
| DIAMOND_VERYSENSITIVE | Yes | --evalue 0.00001 --query-cover 50.0 --very-sensitive |
| DIAMOND_ULTRASENSITIVE | Yes | --evalue 0.00001 --query-cover 50.0 --ultra-sensitive |
| MMSEQS2 |
| MMSEQS2_S1DOT0 | Yes | --e-profile 0.00001 --cov-mode 1 -c 0.50 -s 1.0 |
| MMSEQS2_S2DOT5 | Yes | --e-profile 0.00001 --cov-mode 1 -c 0.50 -s 2.5 |
| MMSEQS2_S6DOT0 | Yes | --e-profile 0.00001 --cov-mode 1 -c 0.50 -s 6.0 |
| MMSEQS2_S7DOT5 | Yes | --e-profile 0.00001 --cov-mode 1 -c 0.50 -s 7.5 |
