# Supplementary material for: Fast and robust estimate of bacterial genus novelty using the percentage of conserved proteins with unique matches (POCPu)
Source: PeerJ. 2025 Nov 14;13:e20259. doi: 10.7717/peerj.20259 (PMC12622232; doi:10.7717/peerj.20259)
Supplement: Supplementary file 6 [file peerj-13-20259-s003.odt]

| Method | Time | Memory | CPU | Disk usage (I/O) |
| --- | --- | --- | --- | --- |
| BLAST |
| BLASTP | 1.000 | 1.000 | 1.000 | 1.000 |
| BLASTPDB | 0.429 | 6.978 | 3.991 | 0.640 |
| DIAMOND |
| DIAMOND_FAST | 0.000 | 4.142 | 4.196 | 1.644 |
| DIAMOND_SENSITIVE | 0.062 | 10.095 | 4.498 | 1.902 |
| DIAMOND_VERYSENSITIVE | 0.043 | 9.844 | 5.178 | 2.290 |
| DIAMOND_ULTRASENSITIVE | 0.156 | 29.795 | 4.901 | 4.943 |
| MMSEQS2 |
| MMSEQS2_S1DOT0 | 0.067 | 7.600 | 4.520 | 0.310 |
| MMSEQS2_S2DOT5 | 0.071 | 7.747 | 4.623 | 0.405 |
| MMSEQS2_S6DOT0 | 0.140 | 16.270 | 5.292 | 0.837 |
| MMSEQS2_S7DOT5 | 0.314 | 16.281 | 5.675 | 1.582 |
